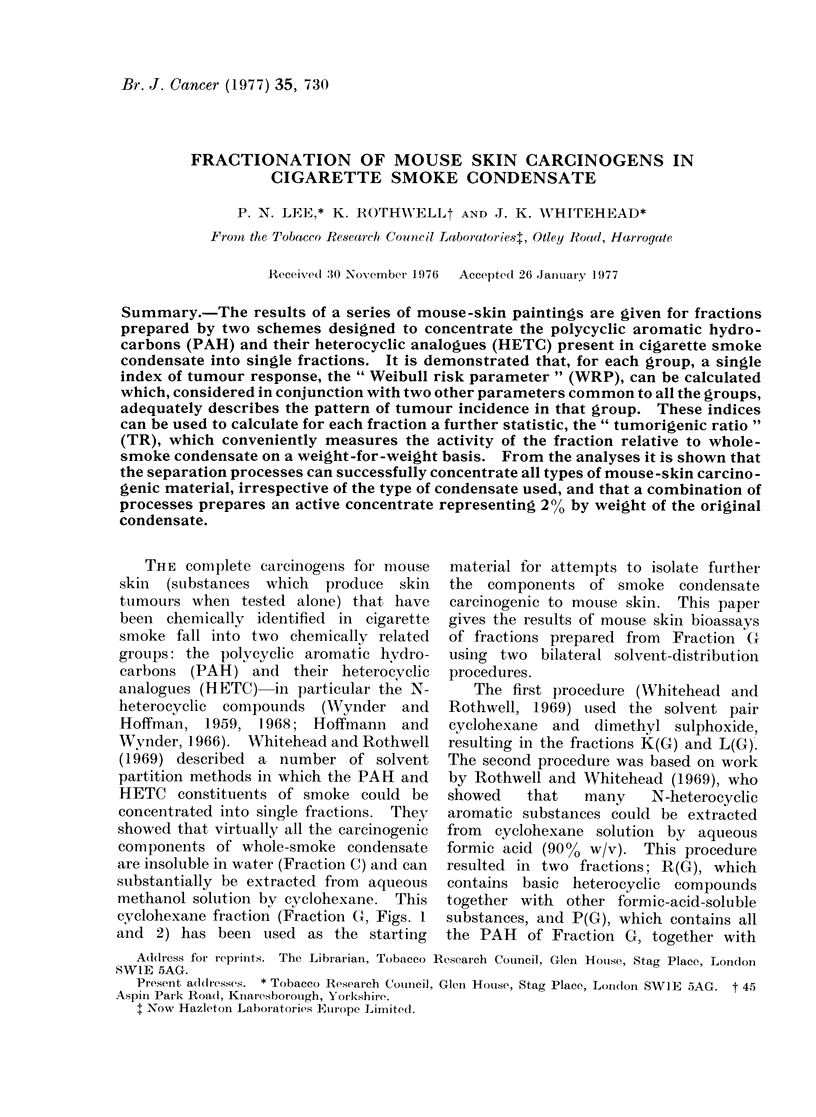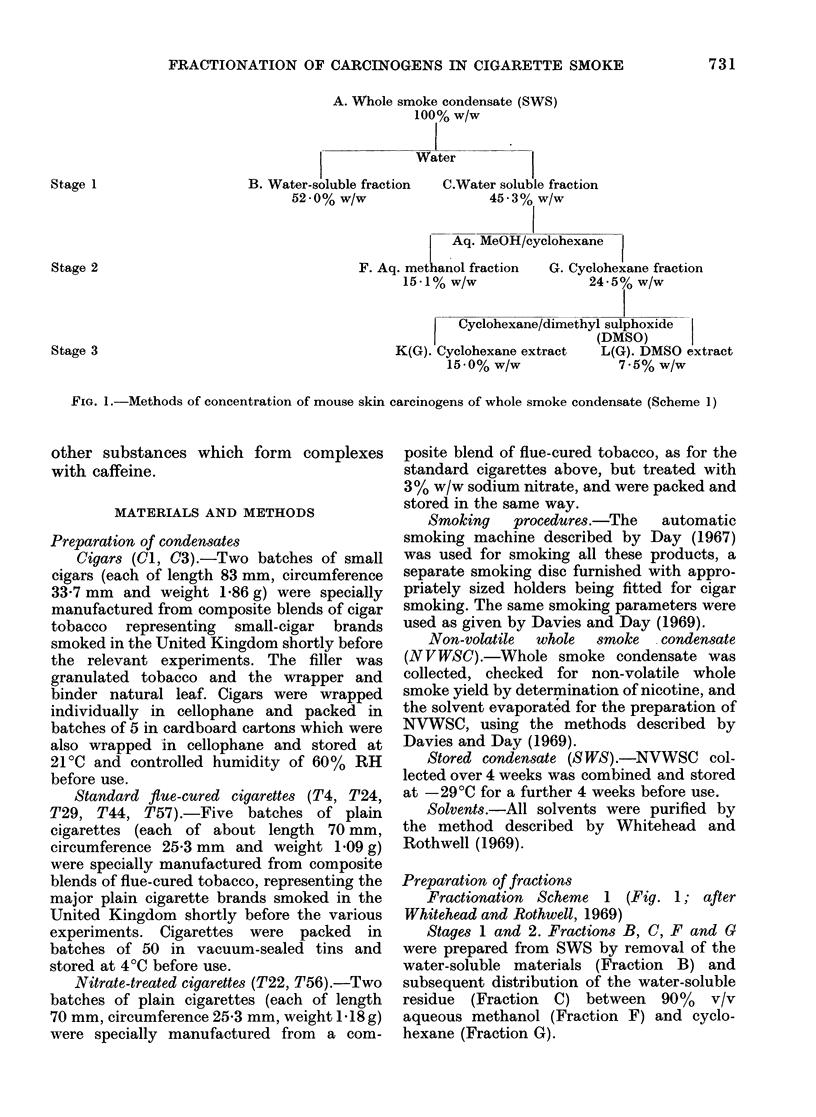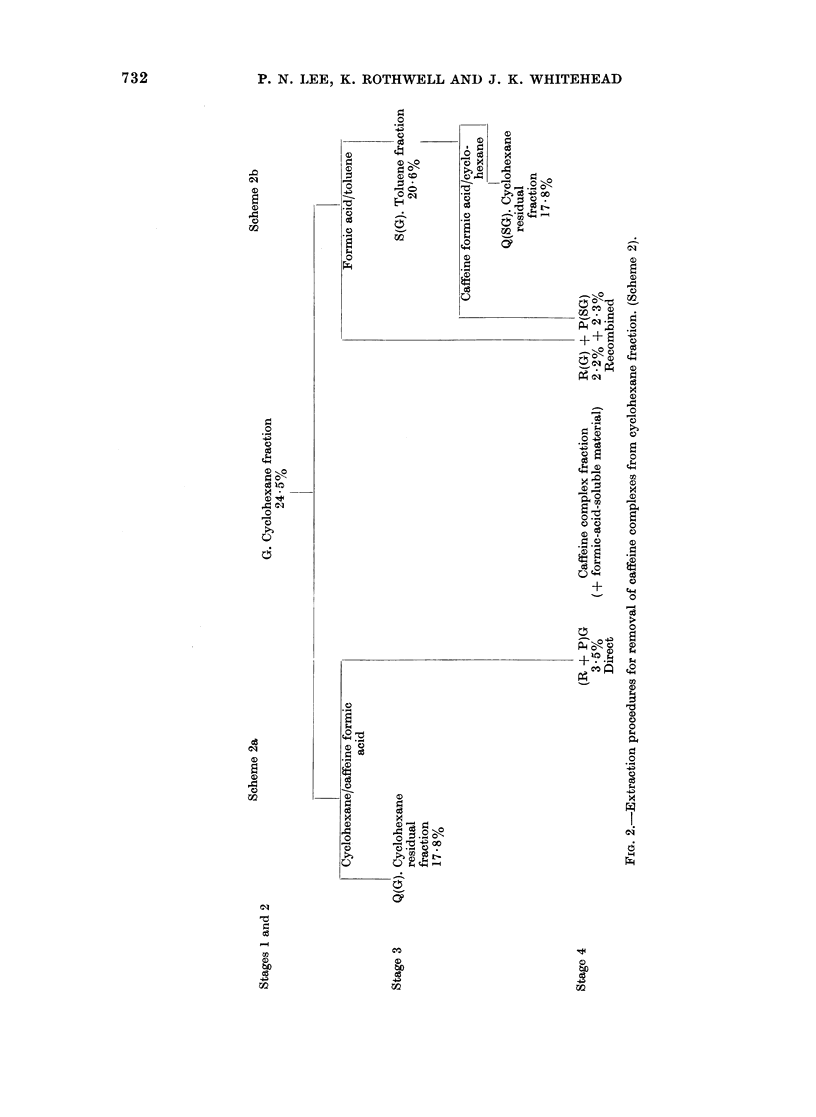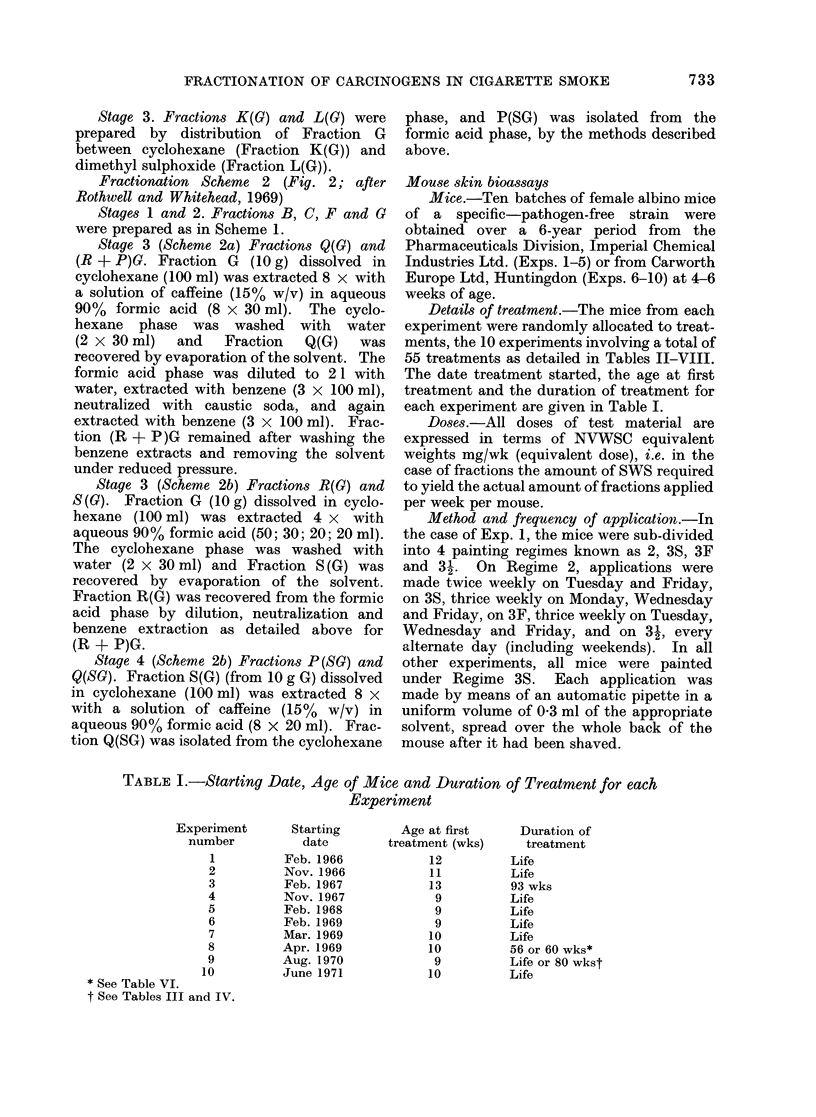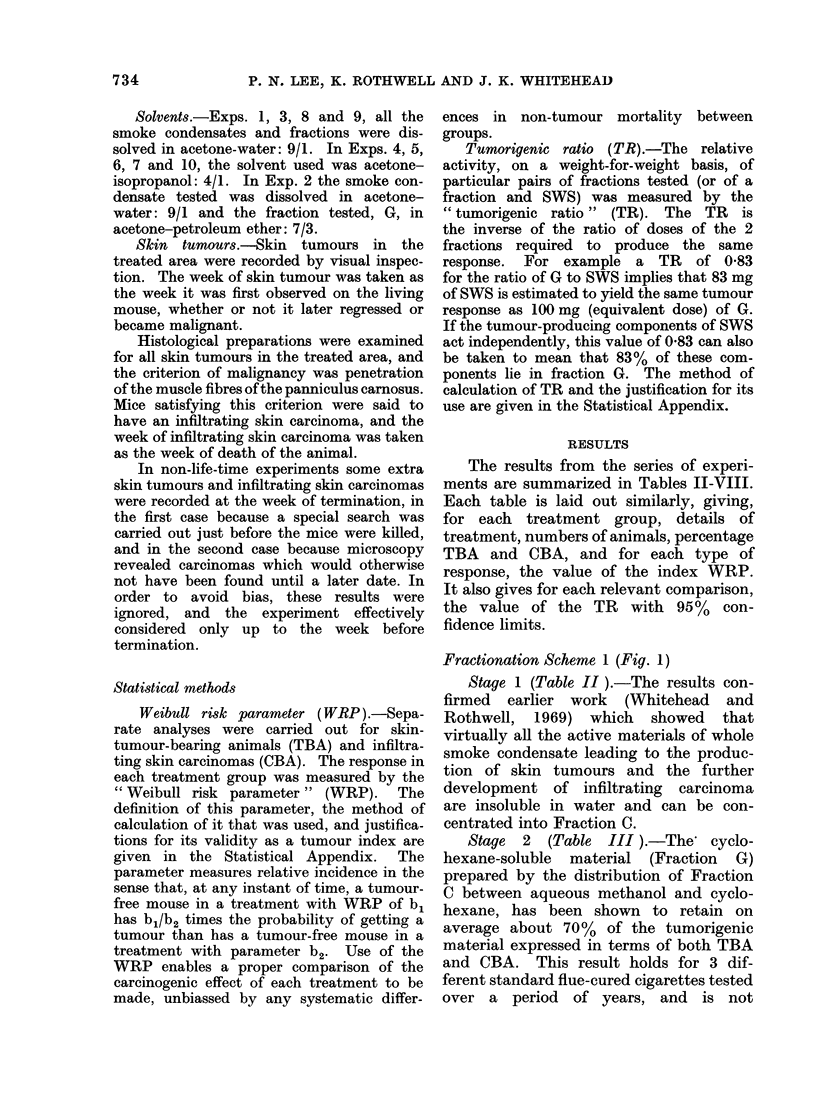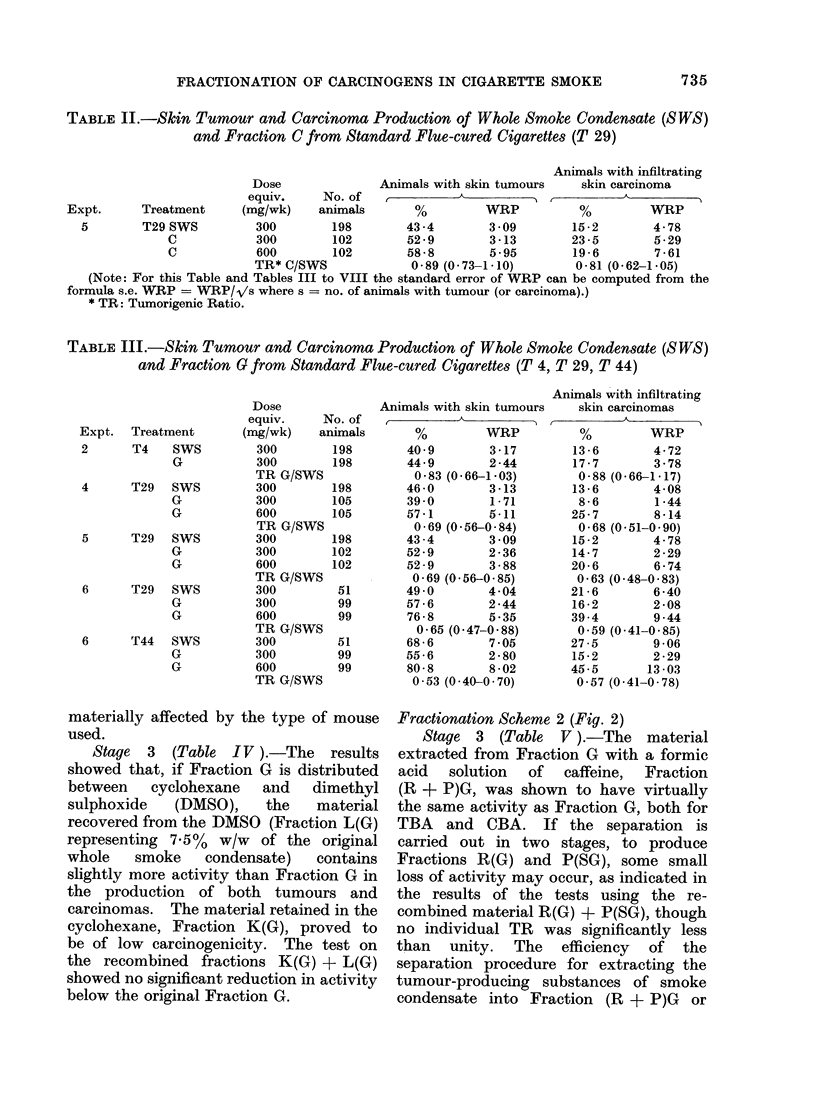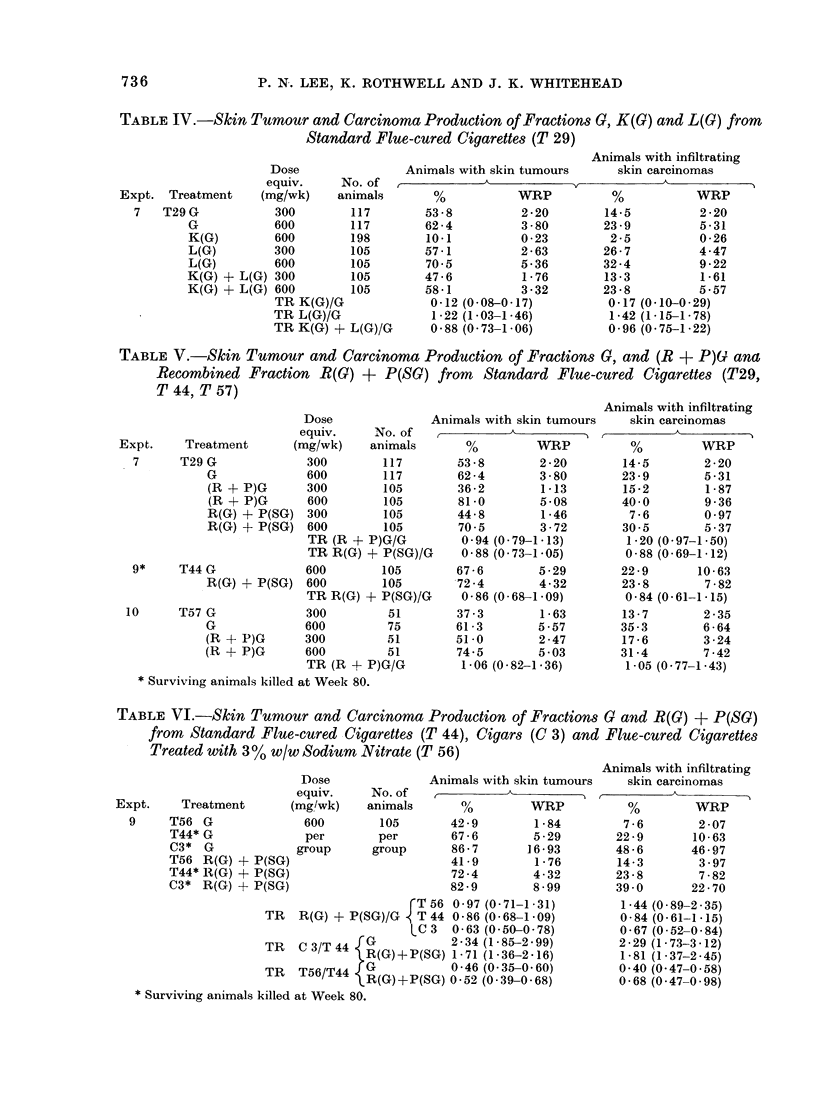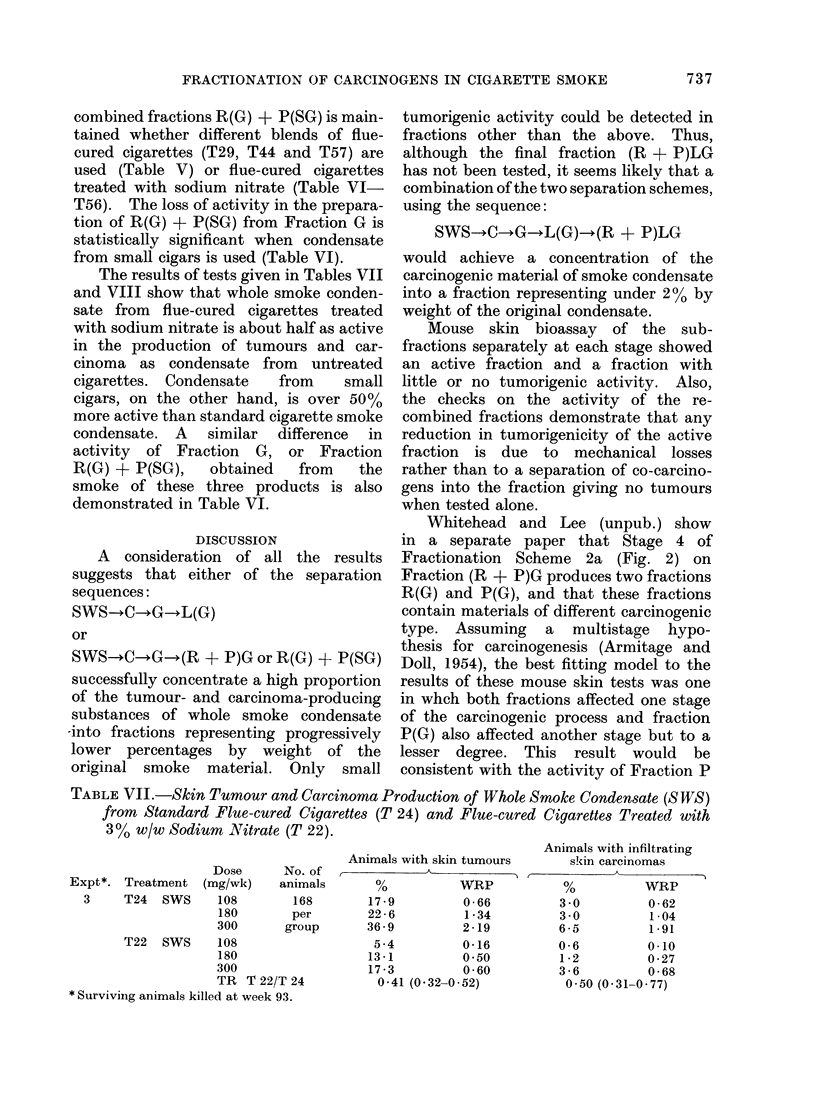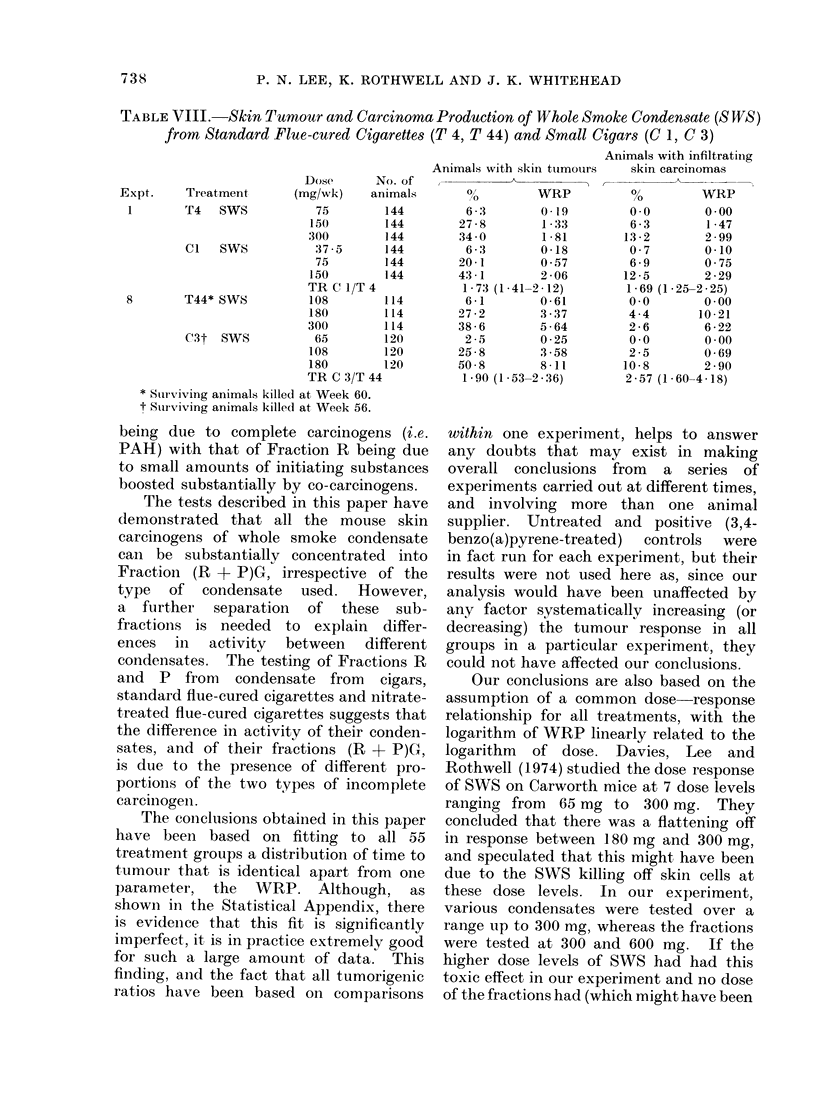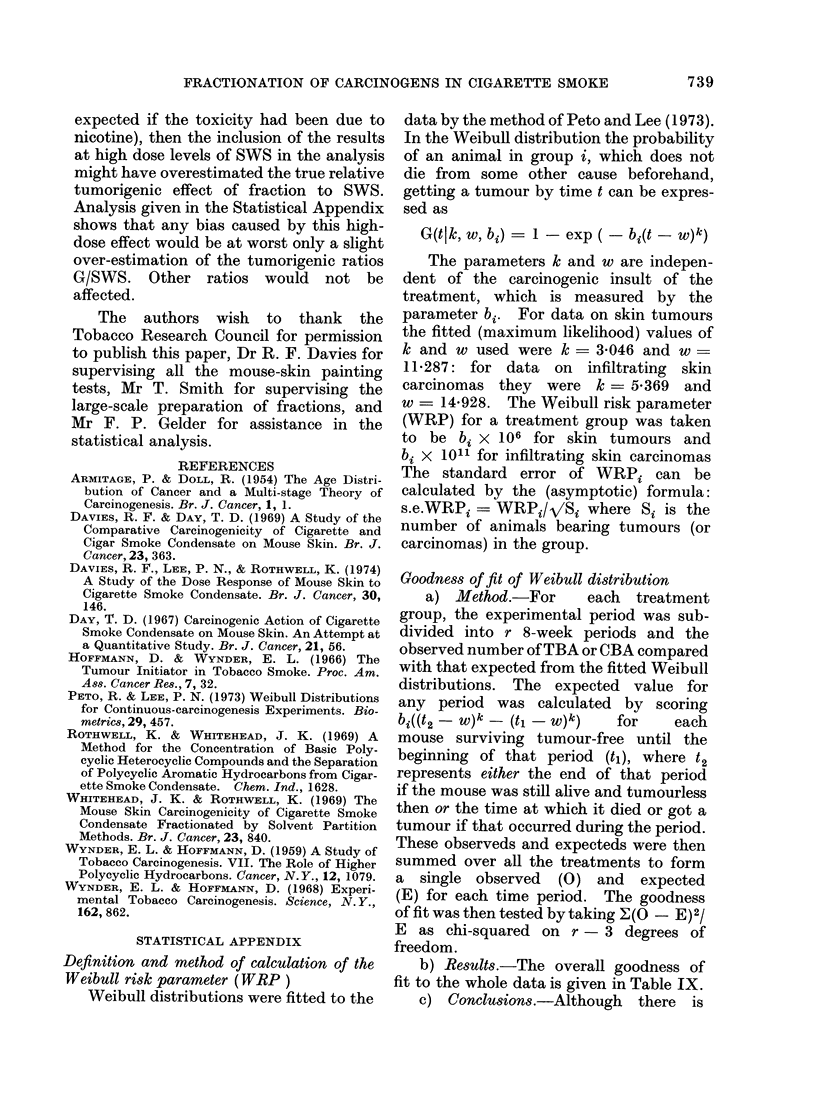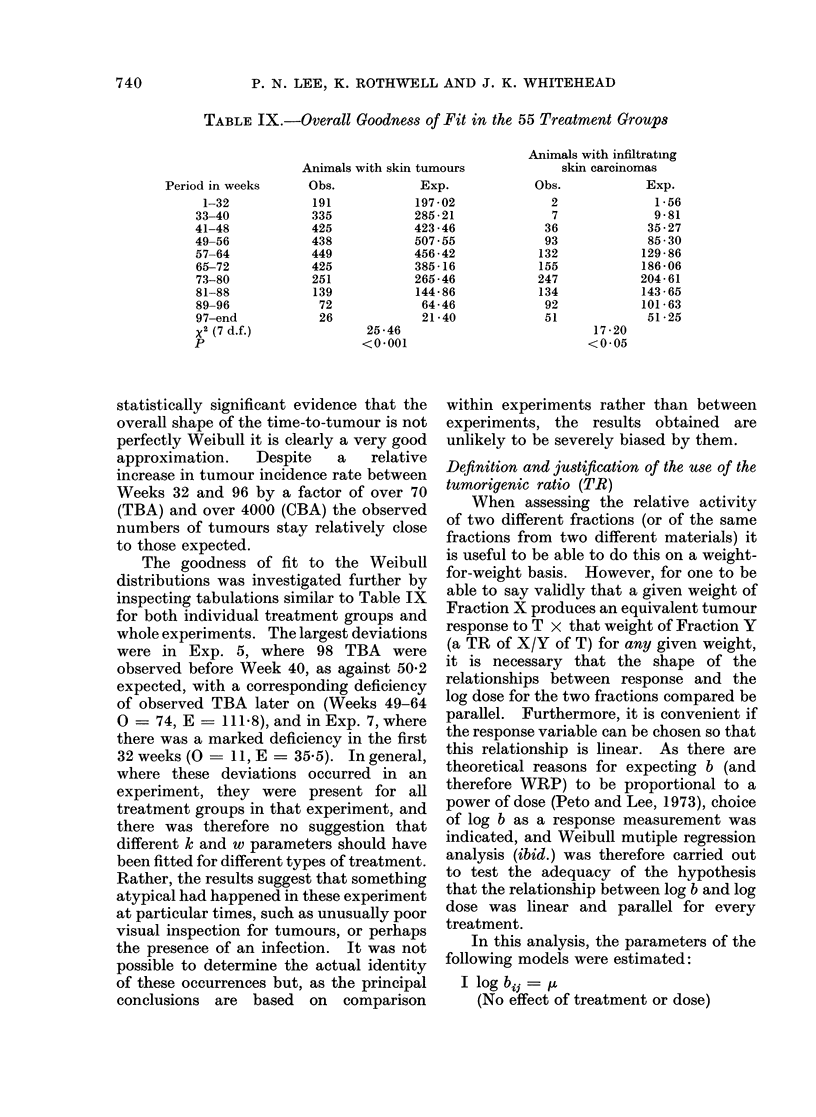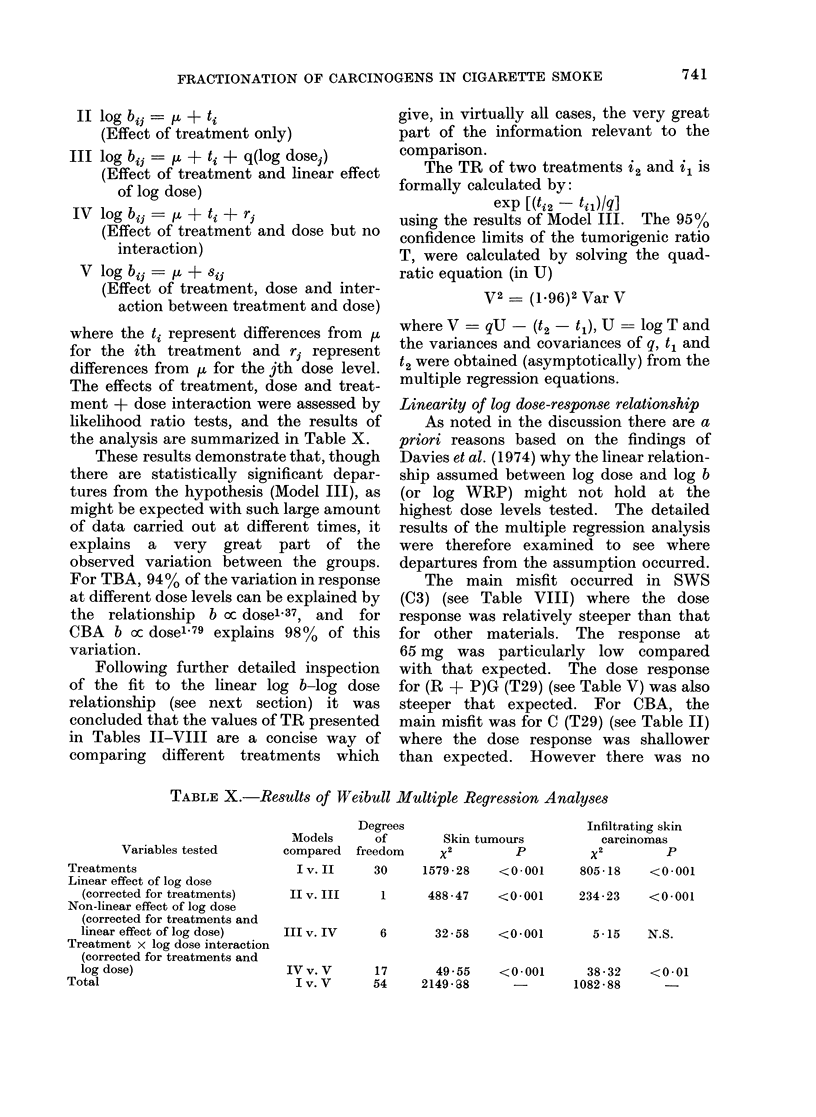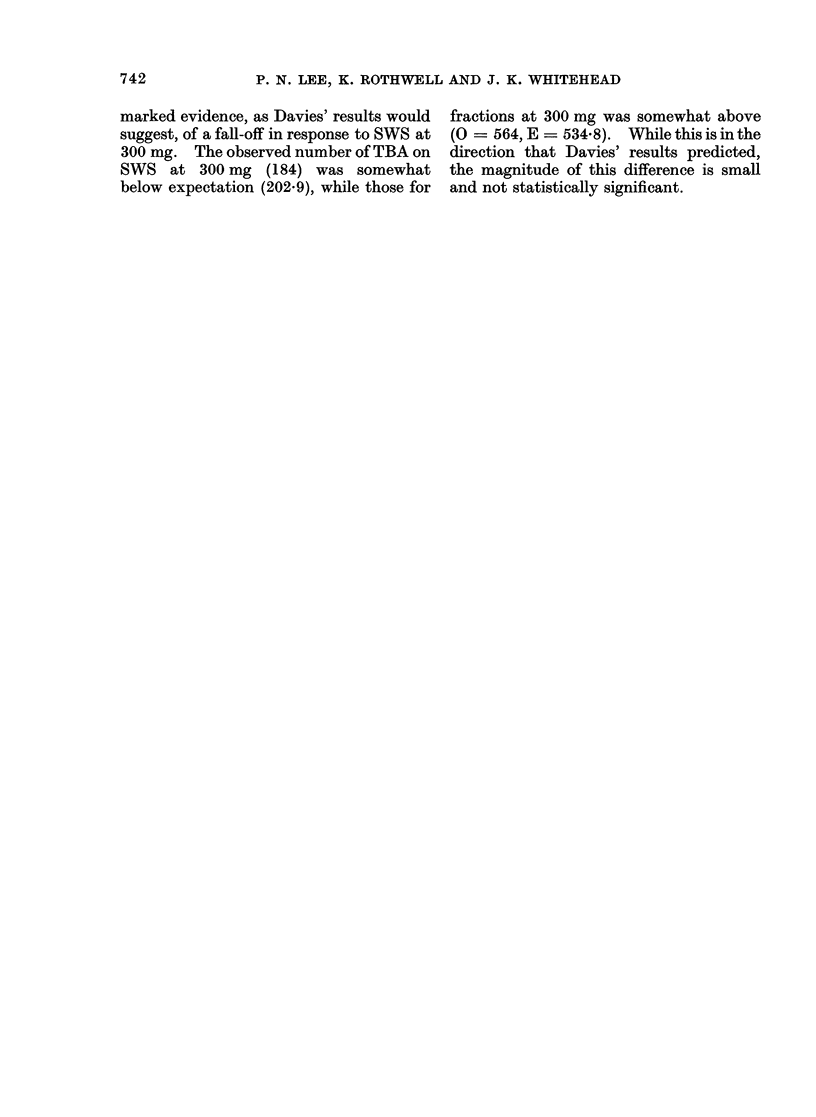# Fractionation of mouse skin carcinogens in cagarette smoke condensate.

**DOI:** 10.1038/bjc.1977.114

**Published:** 1977-06

**Authors:** P. N. Lee, K. Rothwell, J. K. Whitehead

## Abstract

The results of a series of mouse-skin paintings are given for fractions prepared by two schemes designed to concentrate the polycyclic aromatic hydrocarbons (PAH) and their heterocyclic analogues (HETC) present in cigarette smoke condensate into single fractions. It is demonstrated that, for each group, a single index of tumour response, the "Weibull risk parameter" (WRP), can be calculated which, considered in conjunction with two other parameters common to all the groups, adequately describes the pattern of tumour incidence in that group. These indices can be used to calculate for each fraction a further statistic, the "tumorigenic ratio" (TR), which conveniently measures the activity of the fraction relative to whole-smoke condensate on a weight-for-weight basis. From the analyses it is shown that the separation processes can successfully concentrate all types of mouse-skin carcinogenic material, irrespective of the type of condensate used, and that a combination of processes prepares an active concentrate representing 2% by weight of the original condensate.


*Br. J. Cancer* (1977) **35**, 730

### FRACTIONATION OF MOUSE SKIN CARCINOGENS IN CIGARETTE SMOKE CONDENSATE

P. N. LEE,* K. ROTHWELL† AND J. K. WHITEHEAD*

*From the Tobacco Research Council Laboratories‡, Otley Road, Harrogate*

Received 30 November 1976 Accepted 26 January 1977

**Summary.—The results of a series of mouse-skin paintings are given for fractions prepared by two schemes designed to concentrate the polycyclic aromatic hydrocarbons (PAH) and their heterocyclic analogues (HETC) present in cigarette smoke condensate into single fractions. It is demonstrated that, for each group, a single index of tumour response, the " Weibull risk parameter " (WRP), can be calculated which, considered in conjunction with two other parameters common to all the groups, adequately describes the pattern of tumour incidence in that group. These indices can be used to calculate for each fraction a further statistic, the " tumorigenic ratio " (TR), which conveniently measures the activity of the fraction relative to whole-smoke condensate on a weight-for-weight basis. From the analyses it is shown that the separation processes can successfully concentrate all types of mouse-skin carcinogenic material, irrespective of the type of condensate used, and that a combination of processes prepares an active concentrate representing 2% by weight of the original condensate.**

THE complete carcinogens for mouse skin (substances which produce skin tumours when tested alone) that have been chemically identified in cigarette smoke fall into two chemically related groups: the polycyclic aromatic hydrocarbons (PAH) and their heterocyclic analogues (HETC)—in particular the N-heterocyclic compounds (Wynder and Hoffman, 1959, 1968; Hoffmann and Wynder, 1966). Whitehead and Rothwell (1969) described a number of solvent partition methods in which the PAH and HETC constituents of smoke could be concentrated into single fractions. They showed that virtually all the carcinogenic components of whole-smoke condensate are insoluble in water (Fraction C) and can substantially be extracted from aqueous methanol solution by cyclohexane. This cyclohexane fraction (Fraction G, Figs. 1 and 2) has been used as the starting material for attempts to isolate further the components of smoke condensate carcinogenic to mouse skin. This paper gives the results of mouse skin bioassays of fractions prepared from Fraction G using two bilateral solvent-distribution procedures.

The first procedure (Whitehead and Rothwell, 1969) used the solvent pair cyclohexane and dimethyl sulphoxide, resulting in the fractions K(G) and L(G). The second procedure was based on work by Rothwell and Whitehead (1969), who showed that many N-heterocyclic aromatic substances could be extracted from cyclohexane solution by aqueous formic acid (90% w/v). This procedure resulted in two fractions; R(G), which contains basic heterocyclic compounds together with other formic-acid-soluble substances, and P(G), which contains all the PAH of Fraction G, together with

Address for reprints. The Librarian, Tobacco Research Council, Glen House, Stag Place, London SW1E 5AG.

Present addresses. * Tobacco Research Council, Glen House, Stag Place, London SW1E 5AG. † 45 Aspin Park Road, Knaresborough, Yorkshire.

‡ Now Hazleton Laboratories Europe Limited.

FIG. 1.—Methods of concentration of mouse skin carcinogens of whole smoke condensate (Scheme 1)

other substances which form complexes with caffeine.

### MATERIALS AND METHODS

*Preparation of condensates*

*Cigars* (*C*1, *C*3).—Two batches of small cigars (each of length 83 mm, circumference 33·7 mm and weight 1·86 g) were specially manufactured from composite blends of cigar tobacco representing small-cigar brands smoked in the United Kingdom shortly before the relevant experiments. The filler was granulated tobacco and the wrapper and binder natural leaf. Cigars were wrapped individually in cellophane and packed in batches of 5 in cardboard cartons which were also wrapped in cellophane and stored at 21°C and controlled humidity of 60% RH before use.

*Standard flue-cured cigarettes* (*T*4, *T*24, *T*29, *T*44, *T*57).—Five batches of plain cigarettes (each of about length 70 mm, circumference 25·3 mm and weight 1·09 g) were specially manufactured from composite blends of flue-cured tobacco, representing the major plain cigarette brands smoked in the United Kingdom shortly before the various experiments. Cigarettes were packed in batches of 50 in vacuum-sealed tins and stored at 4°C before use.

*Nitrate-treated cigarettes* (*T*22, *T*56).—Two batches of plain cigarettes (each of length 70 mm, circumference 25·3 mm, weight 1·18 g) were specially manufactured from a composite blend of flue-cured tobacco, as for the standard cigarettes above, but treated with 3% w/w sodium nitrate, and were packed and stored in the same way.

*Smoking procedures*.—The automatic smoking machine described by Day (1967) was used for smoking all these products, a separate smoking disc furnished with appropriately sized holders being fitted for cigar smoking. The same smoking parameters were used as given by Davies and Day (1969).

*Non-volatile whole smoke condensate* (*NVWSC*).—Whole smoke condensate was collected, checked for non-volatile whole smoke yield by determination of nicotine, and the solvent evaporated for the preparation of NVWSC, using the methods described by Davies and Day (1969).

*Stored condensate* (*SWS*).—NVWSC collected over 4 weeks was combined and stored at −29°C for a further 4 weeks before use.

*Solvents*.—All solvents were purified by the method described by Whitehead and Rothwell (1969).

*Preparation of fractions*

*Fractionation Scheme* 1 (*Fig.* 1; *after Whitehead and Rothwell*, 1969)

*Stages* 1 *and* 2. *Fractions B*, *C*, *F and G* were prepared from SWS by removal of the water-soluble materials (Fraction B) and subsequent distribution of the water-soluble residue (Fraction C) between 90% v/v aqueous methanol (Fraction F) and cyclohexane (Fraction G).

FIG. 2.—Extraction procedures for removal of caffeine complexes from cyclohexane fraction. (Scheme 2).

*Stage* 3. *Fractions K(G) and L(G)* were prepared by distribution of Fraction G between cyclohexane (Fraction K(G)) and dimethyl sulphoxide (Fraction L(G)).

*Fractionation Scheme* 2 (*Fig.* 2; *after Rothwell and Whitehead*, 1969)

*Stages* 1 *and* 2. *Fractions B, C, F and G* were prepared as in Scheme 1.

*Stage* 3 (*Scheme* 2*a*) *Fractions Q(G) and (R + P)G*. Fraction G (10 g) dissolved in cyclohexane (100 ml) was extracted 8 × with a solution of caffeine (15% w/v) in aqueous 90% formic acid (8 × 30 ml). The cyclohexane phase was washed with water (2 × 30 ml) and Fraction Q(G) was recovered by evaporation of the solvent. The formic acid phase was diluted to 2 l with water, extracted with benzene (3 × 100 ml), neutralized with caustic soda, and again extracted with benzene (3 × 100 ml). Fraction (R + P)G remained after washing the benzene extracts and removing the solvent under reduced pressure.

*Stage* 3 (*Scheme* 2*b*) *Fractions R(G) and S(G)*. Fraction G (10 g) dissolved in cyclohexane (100 ml) was extracted 4 × with aqueous 90% formic acid (50; 30; 20; 20 ml). The cyclohexane phase was washed with water (2 × 30 ml) and Fraction S(G) was recovered by evaporation of the solvent. Fraction R(G) was recovered from the formic acid phase by dilution, neutralization and benzene extraction as detailed above for (R + P)G.

*Stage* 4 (*Scheme* 2*b*) *Fractions P(SG) and Q(SG)*. Fraction S(G) (from 10 g G) dissolved in cyclohexane (100 ml) was extracted 8 × with a solution of caffeine (15% w/v) in aqueous 90% formic acid (8 × 20 ml). Fraction Q(SG) was isolated from the cyclohexane phase, and P(SG) was isolated from the formic acid phase, by the methods described above.

*Mouse skin bioassays*

*Mice*.—Ten batches of female albino mice of a specific—pathogen-free strain were obtained over a 6-year period from the Pharmaceuticals Division, Imperial Chemical Industries Ltd. (Exps. 1–5) or from Carworth Europe Ltd, Huntingdon (Exps. 6–10) at 4–6 weeks of age.

*Details of treatment*.—The mice from each experiment were randomly allocated to treatments, the 10 experiments involving a total of 55 treatments as detailed in Tables II–VIII. The date treatment started, the age at first treatment and the duration of treatment for each experiment are given in Table I.

*Doses*.—All doses of test material are expressed in terms of NVWSC equivalent weights mg/wk (equivalent dose), *i.e.* in the case of fractions the amount of SWS required to yield the actual amount of fractions applied per week per mouse.

*Method and frequency of application*.—In the case of Exp. 1, the mice were sub-divided into 4 painting regimes known as 2, 3S, 3F and 3½. On Regime 2, applications were made twice weekly on Tuesday and Friday, on 3S, thrice weekly on Monday, Wednesday and Friday, on 3F, thrice weekly on Tuesday, Wednesday and Friday, and on 3½, every alternate day (including weekends). In all other experiments, all mice were painted under Regime 3S. Each application was made by means of an automatic pipette in a uniform volume of 0·3 ml of the appropriate solvent, spread over the whole back of the mouse after it had been shaved.

TABLE I.—*Starting Date, Age of Mice and Duration of Treatment for each Experiment*

| Experiment number | Starting date | Age at first treatment (wks) | Duration of treatment |
|---|---|---|---|
| 1 | Feb. 1966 | 12 | Life |
| 2 | Nov. 1966 | 11 | Life |
| 3 | Feb. 1967 | 13 | 93 wks |
| 4 | Nov. 1967 | 9 | Life |
| 5 | Feb. 1968 | 9 | Life |
| 6 | Feb. 1969 | 9 | Life |
| 7 | Mar. 1969 | 10 | Life |
| 8 | Apr. 1969 | 10 | 56 or 60 wks* |
| 9 | Aug. 1970 | 9 | Life or 80 wks† |
| 10 | June 1971 | 10 | Life |

* See Table VI.

† See Tables III and IV.

*Solvents*.—Exps. 1, 3, 8 and 9, all the smoke condensates and fractions were dissolved in acetone-water: 9/1. In Exps. 4, 5, 6, 7 and 10, the solvent used was acetone–isopropanol: 4/1. In Exp. 2 the smoke condensate tested was dissolved in acetone–water: 9/1 and the fraction tested, G, in acetone–petroleum ether: 7/3.

*Skin tumours*.—Skin tumours in the treated area were recorded by visual inspection. The week of skin tumour was taken as the week it was first observed on the living mouse, whether or not it later regressed or became malignant.

Histological preparations were examined for all skin tumours in the treated area, and the criterion of malignancy was penetration of the muscle fibres of the panniculus carnosus. Mice satisfying this criterion were said to have an infiltrating skin carcinoma, and the week of infiltrating skin carcinoma was taken as the week of death of the animal.

In non-life-time experiments some extra skin tumours and infiltrating skin carcinomas were recorded at the week of termination, in the first case because a special search was carried out just before the mice were killed, and in the second case because microscopy revealed carcinomas which would otherwise not have been found until a later date. In order to avoid bias, these results were ignored, and the experiment effectively considered only up to the week before termination.

### *Statistical methods*

*Weibull risk parameter* (*WRP*).—Separate analyses were carried out for skin-tumour-bearing animals (TBA) and infiltrating skin carcinomas (CBA). The response in each treatment group was measured by the " Weibull risk parameter " (WRP). The definition of this parameter, the method of calculation of it that was used, and justifications for its validity as a tumour index are given in the Statistical Appendix. The parameter measures relative incidence in the sense that, at any instant of time, a tumour-free mouse in a treatment with WRP of $b_1$ has $b_1/b_2$ times the probability of getting a tumour than has a tumour-free mouse in a treatment with parameter $b_2$. Use of the WRP enables a proper comparison of the carcinogenic effect of each treatment to be made, unbiassed by any systematic differences in non-tumour mortality between groups.

*Tumorigenic ratio* (*TR*).—The relative activity, on a weight-for-weight basis, of particular pairs of fractions tested (or of a fraction and SWS) was measured by the " tumorigenic ratio " (TR). The TR is the inverse of the ratio of doses of the 2 fractions required to produce the same response. For example a TR of 0·83 for the ratio of G to SWS implies that 83 mg of SWS is estimated to yield the same tumour response as 100 mg (equivalent dose) of G. If the tumour-producing components of SWS act independently, this value of 0·83 can also be taken to mean that 83% of these components lie in fraction G. The method of calculation of TR and the justification for its use are given in the Statistical Appendix.

### RESULTS

The results from the series of experiments are summarized in Tables II-VIII. Each table is laid out similarly, giving, for each treatment group, details of treatment, numbers of animals, percentage TBA and CBA, and for each type of response, the value of the index WRP. It also gives for each relevant comparison, the value of the TR with 95% confidence limits.

### *Fractionation Scheme* 1 (*Fig*. 1)

*Stage* 1 (*Table II* ).—The results confirmed earlier work (Whitehead and Rothwell, 1969) which showed that virtually all the active materials of whole smoke condensate leading to the production of skin tumours and the further development of infiltrating carcinoma are insoluble in water and can be concentrated into Fraction C.

*Stage* 2 (*Table III* ).—The cyclohexane-soluble material (Fraction G) prepared by the distribution of Fraction C between aqueous methanol and cyclohexane, has been shown to retain on average about 70% of the tumorigenic material expressed in terms of both TBA and CBA. This result holds for 3 different standard flue-cured cigarettes tested over a period of years, and is not

TABLE II.—*Skin Tumour and Carcinoma Production of Whole Smoke Condensate (SWS) and Fraction C from Standard Flue-cured Cigarettes (T 29)*

| Expt. | Treatment | Dose equiv. (mg/wk) | No. of animals | Animals with skin tumours % | Animals with skin tumours WRP | Animals with infiltrating skin carcinoma % | Animals with infiltrating skin carcinoma WRP |
|---|---|---|---|---|---|---|---|
| 5 | T29 SWS | 300 | 198 | 43·4 | 3·09 | 15·2 | 4·78 |
| | C | 300 | 102 | 52·9 | 3·13 | 23·5 | 5·29 |
| | C | 600 | 102 | 58·8 | 5·95 | 19·6 | 7·61 |
| | | TR* C/SWS | | 0·89 (0·73–1·10) | | 0·81 (0·62–1·05) | |

(Note: For this Table and Tables III to VIII the standard error of WRP can be computed from the formula s.e. WRP = WRP/$\sqrt{s}$ where s = no. of animals with tumour (or carcinoma).)

* TR: Tumorigenic Ratio.

TABLE III.—*Skin Tumour and Carcinoma Production of Whole Smoke Condensate (SWS) and Fraction G from Standard Flue-cured Cigarettes (T 4, T 29, T 44)*

| Expt. | Treatment | | Dose equiv. (mg/wk) | No. of animals | Animals with skin tumours % | Animals with skin tumours WRP | Animals with infiltrating skin carcinomas % | Animals with infiltrating skin carcinomas WRP |
|---|---|---|---|---|---|---|---|---|
| 2 | T4 | SWS | 300 | 198 | 40·9 | 3·17 | 13·6 | 4·72 |
| | | G | 300 | 198 | 44·9 | 2·44 | 17·7 | 3·78 |
| | | | TR G/SWS | | 0·83 (0·66–1·03) | | 0·88 (0·66–1·17) | |
| 4 | T29 | SWS | 300 | 198 | 46·0 | 3·13 | 13·6 | 4·08 |
| | | G | 300 | 105 | 39·0 | 1·71 | 8·6 | 1·44 |
| | | G | 600 | 105 | 57·1 | 5·11 | 25·7 | 8·14 |
| | | | TR G/SWS | | 0·69 (0·56–0·84) | | 0·68 (0·51–0·90) | |
| 5 | T29 | SWS | 300 | 198 | 43·4 | 3·09 | 15·2 | 4·78 |
| | | G | 300 | 102 | 52·9 | 2·36 | 14·7 | 2·29 |
| | | G | 600 | 102 | 52·9 | 3·88 | 20·6 | 6·74 |
| | | | TR G/SWS | | 0·69 (0·56–0·85) | | 0·63 (0·48–0·83) | |
| 6 | T29 | SWS | 300 | 51 | 49·0 | 4·04 | 21·6 | 6·40 |
| | | G | 300 | 99 | 57·6 | 2·44 | 16·2 | 2·08 |
| | | G | 600 | 99 | 76·8 | 5·35 | 39·4 | 9·44 |
| | | | TR G/SWS | | 0·65 (0·47–0·88) | | 0·59 (0·41–0·85) | |
| 6 | T44 | SWS | 300 | 51 | 68·6 | 7·05 | 27·5 | 9·06 |
| | | G | 300 | 99 | 55·6 | 2·80 | 15·2 | 2·29 |
| | | G | 600 | 99 | 80·8 | 8·02 | 45·5 | 13·03 |
| | | | TR G/SWS | | 0·53 (0·40–0·70) | | 0·57 (0·41–0·78) | |

materially affected by the type of mouse used.

*Stage* 3 (*Table IV*).—The results showed that, if Fraction G is distributed between cyclohexane and dimethyl sulphoxide (DMSO), the material recovered from the DMSO (Fraction L(G) representing 7·5% w/w of the original whole smoke condensate) contains slightly more activity than Fraction G in the production of both tumours and carcinomas. The material retained in the cyclohexane, Fraction K(G), proved to be of low carcinogenicity. The test on the recombined fractions K(G) + L(G) showed no significant reduction in activity below the original Fraction G.

*Fractionation Scheme* 2 (*Fig.* 2)

*Stage* 3 (*Table V*).—The material extracted from Fraction G with a formic acid solution of caffeine, Fraction (R + P)G, was shown to have virtually the same activity as Fraction G, both for TBA and CBA. If the separation is carried out in two stages, to produce Fractions R(G) and P(SG), some small loss of activity may occur, as indicated in the results of the tests using the recombined material R(G) + P(SG), though no individual TR was significantly less than unity. The efficiency of the separation procedure for extracting the tumour-producing substances of smoke condensate into Fraction (R + P)G or

TABLE IV.—*Skin Tumour and Carcinoma Production of Fractions G, K(G) and L(G) from Standard Flue-cured Cigarettes (T 29)*

| Expt. | Treatment | Dose equiv. (mg/wk) | No. of animals | Animals with skin tumours % | Animals with skin tumours WRP | Animals with infiltrating skin carcinomas % | Animals with infiltrating skin carcinomas WRP |
|---|---|---|---|---|---|---|---|
| 7 | T29 G | 300 | 117 | 53·8 | 2·20 | 14·5 | 2·20 |
| | G | 600 | 117 | 62·4 | 3·80 | 23·9 | 5·31 |
| | K(G) | 600 | 198 | 10·1 | 0·23 | 2·5 | 0·26 |
| | L(G) | 300 | 105 | 57·1 | 2·63 | 26·7 | 4·47 |
| | L(G) | 600 | 105 | 70·5 | 5·36 | 32·4 | 9·22 |
| | K(G) + L(G) | 300 | 105 | 47·6 | 1·76 | 13·3 | 1·61 |
| | K(G) + L(G) | 600 | 105 | 58·1 | 3·32 | 23·8 | 5·57 |
| | | TR K(G)/G | | 0·12 (0·08–0·17) | | 0·17 (0·10–0·29) | |
| | | TR L(G)/G | | 1·22 (1·03–1·46) | | 1·42 (1·15–1·78) | |
| | | TR K(G) + L(G)/G | | 0·88 (0·73–1·06) | | 0·96 (0·75–1·22) | |

TABLE V.—*Skin Tumour and Carcinoma Production of Fractions G, and (R + P)G and Recombined Fraction R(G) + P(SG) from Standard Flue-cured Cigarettes (T29, T 44, T 57)*

| Expt. | Treatment | Dose equiv. (mg/wk) | No. of animals | Animals with skin tumours % | Animals with skin tumours WRP | Animals with infiltrating skin carcinomas % | Animals with infiltrating skin carcinomas WRP |
|---|---|---|---|---|---|---|---|
| 7 | T29 G | 300 | 117 | 53·8 | 2·20 | 14·5 | 2·20 |
| | G | 600 | 117 | 62·4 | 3·80 | 23·9 | 5·31 |
| | (R + P)G | 300 | 105 | 36·2 | 1·13 | 15·2 | 1·87 |
| | (R + P)G | 600 | 105 | 81·0 | 5·08 | 40·0 | 9·36 |
| | R(G) + P(SG) | 300 | 105 | 44·8 | 1·46 | 7·6 | 0·97 |
| | R(G) + P(SG) | 600 | 105 | 70·5 | 3·72 | 30·5 | 5·37 |
| | | TR (R + P)G/G | | 0·94 (0·79–1·13) | | 1·20 (0·97–1·50) | |
| | | TR R(G) + P(SG)/G | | 0·88 (0·73–1·05) | | 0·88 (0·69–1·12) | |
| 9* | T44 G | 600 | 105 | 67·6 | 5·29 | 22·9 | 10·63 |
| | R(G) + P(SG) | 600 | 105 | 72·4 | 4·32 | 23·8 | 7·82 |
| | | TR R(G) + P(SG)/G | | 0·86 (0·68–1·09) | | 0·84 (0·61–1·15) | |
| 10 | T57 G | 300 | 51 | 37·3 | 1·63 | 13·7 | 2·35 |
| | G | 600 | 75 | 61·3 | 5·57 | 35·3 | 6·64 |
| | (R + P)G | 300 | 51 | 51·0 | 2·47 | 17·6 | 3·24 |
| | (R + P)G | 600 | 51 | 74·5 | 5·03 | 31·4 | 7·42 |
| | | TR (R + P)G/G | | 1·06 (0·82–1·36) | | 1·05 (0·77–1·43) | |

* Surviving animals killed at Week 80.

TABLE VI.—*Skin Tumour and Carcinoma Production of Fractions G and R(G) + P(SG) from Standard Flue-cured Cigarettes (T 44), Cigars (C 3) and Flue-cured Cigarettes Treated with 3% w/w Sodium Nitrate (T 56)*

| Expt. | Treatment | Dose equiv. (mg/wk) | No. of animals | Animals with skin tumours % | Animals with skin tumours WRP | Animals with infiltrating skin carcinomas % | Animals with infiltrating skin carcinomas WRP |
|---|---|---|---|---|---|---|---|
| 9 | T56 G | 600 | 105 | 42·9 | 1·84 | 7·6 | 2·07 |
| | T44* G | per | per | 67·6 | 5·29 | 22·9 | 10·63 |
| | C3* G | group | group | 86·7 | 16·93 | 48·6 | 46·97 |
| | T56 R(G) + P(SG) | | | 41·9 | 1·76 | 14·3 | 3·97 |
| | T44* R(G) + P(SG) | | | 72·4 | 4·32 | 23·8 | 7·82 |
| | C3* R(G) + P(SG) | | | 82·9 | 8·99 | 39·0 | 22·70 |
| | TR | R(G) + P(SG)/G | T 56 | 0·97 (0·71–1·31) | | 1·44 (0·89–2·35) | |
| | | | T 44 | 0·86 (0·68–1·09) | | 0·84 (0·61–1·15) | |
| | | | C 3 | 0·63 (0·50–0·78) | | 0·67 (0·52–0·84) | |
| | TR | C 3/T 44 | G | 2·34 (1·85–2·99) | | 2·29 (1·73–3·12) | |
| | | | R(G)+P(SG) | 1·71 (1·36–2·16) | | 1·81 (1·37–2·45) | |
| | TR | T56/T44 | G | 0·46 (0·35–0·60) | | 0·40 (0·47–0·58) | |
| | | | R(G)+P(SG) | 0·52 (0·39–0·68) | | 0·68 (0·47–0·98) | |

* Surviving animals killed at Week 80.

combined fractions R(G) + P(SG) is maintained whether different blends of flue-cured cigarettes (T29, T44 and T57) are used (Table V) or flue-cured cigarettes treated with sodium nitrate (Table VI—T56). The loss of activity in the preparation of R(G) + P(SG) from Fraction G is statistically significant when condensate from small cigars is used (Table VI).

The results of tests given in Tables VII and VIII show that whole smoke condensate from flue-cured cigarettes treated with sodium nitrate is about half as active in the production of tumours and carcinoma as condensate from untreated cigarettes. Condensate from small cigars, on the other hand, is over 50% more active than standard cigarette smoke condensate. A similar difference in activity of Fraction G, or Fraction R(G) + P(SG), obtained from the smoke of these three products is also demonstrated in Table VI.

### DISCUSSION

A consideration of all the results suggests that either of the separation sequences:

SWS→C→G→L(G)

or

SWS→C→G→(R + P)G or R(G) + P(SG)

successfully concentrate a high proportion of the tumour- and carcinoma-producing substances of whole smoke condensate into fractions representing progressively lower percentages by weight of the original smoke material. Only small tumorigenic activity could be detected in fractions other than the above. Thus, although the final fraction (R + P)LG has not been tested, it seems likely that a combination of the two separation schemes, using the sequence:

SWS→C→G→L(G)→(R + P)LG

would achieve a concentration of the carcinogenic material of smoke condensate into a fraction representing under 2% by weight of the original condensate.

Mouse skin bioassay of the subfractions separately at each stage showed an active fraction and a fraction with little or no tumorigenic activity. Also, the checks on the activity of the recombined fractions demonstrate that any reduction in tumorigenicity of the active fraction is due to mechanical losses rather than to a separation of co-carcinogens into the fraction giving no tumours when tested alone.

Whitehead and Lee (unpub.) show in a separate paper that Stage 4 of Fractionation Scheme 2a (Fig. 2) on Fraction (R + P)G produces two fractions R(G) and P(G), and that these fractions contain materials of different carcinogenic type. Assuming a multistage hypothesis for carcinogenesis (Armitage and Doll, 1954), the best fitting model to the results of these mouse skin tests was one in whch both fractions affected one stage of the carcinogenic process and fraction P(G) also affected another stage but to a lesser degree. This result would be consistent with the activity of Fraction P

TABLE VII.—*Skin Tumour and Carcinoma Production of Whole Smoke Condensate (SWS) from Standard Flue-cured Cigarettes (T 24) and Flue-cured Cigarettes Treated with 3% w/w Sodium Nitrate (T 22).*

| Expt*. | Treatment | Dose (mg/wk) | No. of animals | Animals with skin tumours % | Animals with skin tumours WRP | Animals with infiltrating skin carcinomas % | Animals with infiltrating skin carcinomas WRP |
|---|---|---|---|---|---|---|---|
| 3 | T24 SWS | 108 | 168 | 17·9 | 0·66 | 3·0 | 0·62 |
| | | 180 | per | 22·6 | 1·34 | 3·0 | 1·04 |
| | | 300 | group | 36·9 | 2·19 | 6·5 | 1·91 |
| | T22 SWS | 108 | | 5·4 | 0·16 | 0·6 | 0·10 |
| | | 180 | | 13·1 | 0·50 | 1·2 | 0·27 |
| | | 300 | | 17·3 | 0·60 | 3·6 | 0·68 |
| | | TR | T 22/T 24 | 0·41 (0·32–0·52) | | 0·50 (0·31–0·77) | |

* Surviving animals killed at week 93.

TABLE VIII.—*Skin Tumour and Carcinoma Production of Whole Smoke Condensate (SWS) from Standard Flue-cured Cigarettes (T 4, T 44) and Small Cigars (C 1, C 3)*

| Expt. | Treatment | Dose (mg/wk) | No. of animals | Animals with skin tumours % | Animals with skin tumours WRP | Animals with infiltrating skin carcinomas % | Animals with infiltrating skin carcinomas WRP |
|---|---|---|---|---|---|---|---|
| 1 | T4 SWS | 75 | 144 | 6·3 | 0·19 | 0·0 | 0·00 |
| | | 150 | 144 | 27·8 | 1·33 | 6·3 | 1·47 |
| | | 300 | 144 | 34·0 | 1·81 | 13·2 | 2·99 |
| | C1 SWS | 37·5 | 144 | 6·3 | 0·18 | 0·7 | 0·10 |
| | | 75 | 144 | 20·1 | 0·57 | 6·9 | 0·75 |
| | | 150 | 144 | 43·1 | 2·06 | 12·5 | 2·29 |
| | | TR C 1/T 4 | | 1·73 (1·41–2·12) | | 1·69 (1·25–2·25) | |
| 8 | T44* SWS | 108 | 114 | 6·1 | 0·61 | 0·0 | 0·00 |
| | | 180 | 114 | 27·2 | 3·37 | 4·4 | 10·21 |
| | | 300 | 114 | 38·6 | 5·64 | 2·6 | 6·22 |
| | C3† SWS | 65 | 120 | 2·5 | 0·25 | 0·0 | 0·00 |
| | | 108 | 120 | 25·8 | 3·58 | 2·5 | 0·69 |
| | | 180 | 120 | 50·8 | 8·11 | 10·8 | 2·90 |
| | | TR C 3/T 44 | | 1·90 (1·53–2·36) | | 2·57 (1·60–4·18) | |

* Surviving animals killed at Week 60.
† Surviving animals killed at Week 56.

being due to complete carcinogens (*i.e.* PAH) with that of Fraction R being due to small amounts of initiating substances boosted substantially by co-carcinogens.

The tests described in this paper have demonstrated that all the mouse skin carcinogens of whole smoke condensate can be substantially concentrated into Fraction (R + P)G, irrespective of the type of condensate used. However, a further separation of these subfractions is needed to explain differences in activity between different condensates. The testing of Fractions R and P from condensate from cigars, standard flue-cured cigarettes and nitrate-treated flue-cured cigarettes suggests that the difference in activity of their condensates, and of their fractions (R + P)G, is due to the presence of different proportions of the two types of incomplete carcinogen.

The conclusions obtained in this paper have been based on fitting to all 55 treatment groups a distribution of time to tumour that is identical apart from one parameter, the WRP. Although, as shown in the Statistical Appendix, there is evidence that this fit is significantly imperfect, it is in practice extremely good for such a large amount of data. This finding, and the fact that all tumorigenic ratios have been based on comparisons *within* one experiment, helps to answer any doubts that may exist in making overall conclusions from a series of experiments carried out at different times, and involving more than one animal supplier. Untreated and positive (3,4-benzo(a)pyrene-treated) controls were in fact run for each experiment, but their results were not used here as, since our analysis would have been unaffected by any factor systematically increasing (or decreasing) the tumour response in all groups in a particular experiment, they could not have affected our conclusions.

Our conclusions are also based on the assumption of a common dose—response relationship for all treatments, with the logarithm of WRP linearly related to the logarithm of dose. Davies, Lee and Rothwell (1974) studied the dose response of SWS on Carworth mice at 7 dose levels ranging from 65 mg to 300 mg. They concluded that there was a flattening off in response between 180 mg and 300 mg, and speculated that this might have been due to the SWS killing off skin cells at these dose levels. In our experiment, various condensates were tested over a range up to 300 mg, whereas the fractions were tested at 300 and 600 mg. If the higher dose levels of SWS had had this toxic effect in our experiment and no dose of the fractions had (which might have been expected if the toxicity had been due to nicotine), then the inclusion of the results at high dose levels of SWS in the analysis might have overestimated the true relative tumorigenic effect of fraction to SWS. Analysis given in the Statistical Appendix shows that any bias caused by this high-dose effect would be at worst only a slight over-estimation of the tumorigenic ratios G/SWS. Other ratios would not be affected.

The authors wish to thank the Tobacco Research Council for permission to publish this paper, Dr R. F. Davies for supervising all the mouse-skin painting tests, Mr T. Smith for supervising the large-scale preparation of fractions, and Mr F. P. Gelder for assistance in the statistical analysis.

### REFERENCES

ARMITAGE, P. & DOLL, R. (1954) The Age Distribution of Cancer and a Multi-stage Theory of Carcinogenesis. *Br. J. Cancer*, **1**, 1.

DAVIES, R. F. & DAY, T. D. (1969) A Study of the Comparative Carcinogenicity of Cigarette and Cigar Smoke Condensate on Mouse Skin. *Br. J. Cancer*, **23**, 363.

DAVIES, R. F., LEE, P. N., & ROTHWELL, K. (1974) A Study of the Dose Response of Mouse Skin to Cigarette Smoke Condensate. *Br. J. Cancer*, **30**, 146.

DAY, T. D. (1967) Carcinogenic Action of Cigarette Smoke Condensate on Mouse Skin. An Attempt at a Quantitative Study. *Br. J. Cancer*, **21**, 56.

HOFFMANN, D. & WYNDER, E. L. (1966) The Tumour Initiator in Tobacco Smoke. *Proc. Am. Ass. Cancer Res.*, **7**, 32.

PETO, R. & LEE, P. N. (1973) Weibull Distributions for Continuous-carcinogenesis Experiments. *Biometrics*, **29**, 457.

ROTHWELL, K. & WHITEHEAD, J. K. (1969) A Method for the Concentration of Basic Polycyclic Heterocyclic Compounds and the Separation of Polycyclic Aromatic Hydrocarbons from Cigarette Smoke Condensate. *Chem. Ind.*, 1628.

WHITEHEAD, J. K. & ROTHWELL, K. (1969) The Mouse Skin Carcinogenicity of Cigarette Smoke Condensate Fractionated by Solvent Partition Methods. *Br. J. Cancer*, **23**, 840.

WYNDER, E. L. & HOFFMANN, D. (1959) A Study of Tobacco Carcinogenesis. VII. The Role of Higher Polycyclic Hydrocarbons. *Cancer, N.Y.*, **12**, 1079.

WYNDER, E. L. & HOFFMANN, D. (1968) Experimental Tobacco Carcinogenesis. *Science, N.Y.*, **162**, 862.

### STATISTICAL APPENDIX

### *Definition and method of calculation of the Weibull risk parameter (WRP)*

Weibull distributions were fitted to the data by the method of Peto and Lee (1973). In the Weibull distribution the probability of an animal in group $i$, which does not die from some other cause beforehand, getting a tumour by time $t$ can be expressed as

$$G(t|k, w, b_i) = 1 - \exp(-b_i(t-w)^k)$$

The parameters $k$ and $w$ are independent of the carcinogenic insult of the treatment, which is measured by the parameter $b_i$. For data on skin tumours the fitted (maximum likelihood) values of $k$ and $w$ used were $k = 3{\cdot}046$ and $w = 11{\cdot}287$: for data on infiltrating skin carcinomas they were $k = 5{\cdot}369$ and $w = 14{\cdot}928$. The Weibull risk parameter (WRP) for a treatment group was taken to be $b_i \times 10^6$ for skin tumours and $b_i \times 10^{11}$ for infiltrating skin carcinomas The standard error of $WRP_i$ can be calculated by the (asymptotic) formula: $s.e.WRP_i = WRP_i/\sqrt{S_i}$ where $S_i$ is the number of animals bearing tumours (or carcinomas) in the group.

### *Goodness of fit of Weibull distribution*

a) *Method*.—For each treatment group, the experimental period was subdivided into $r$ 8-week periods and the observed number of TBA or CBA compared with that expected from the fitted Weibull distributions. The expected value for any period was calculated by scoring $b_i((t_2 - w)^k - (t_1 - w)^k)$ for each mouse surviving tumour-free until the beginning of that period ($t_1$), where $t_2$ represents *either* the end of that period if the mouse was still alive and tumourless then *or* the time at which it died or got a tumour if that occurred during the period. These observeds and expecteds were then summed over all the treatments to form a single observed (O) and expected (E) for each time period. The goodness of fit was then tested by taking $\Sigma(O - E)^2/E$ as chi-squared on $r - 3$ degrees of freedom.

b) *Results*.—The overall goodness of fit to the whole data is given in Table IX.

c) *Conclusions*.—Although there is

TABLE IX.—*Overall Goodness of Fit in the 55 Treatment Groups*

| Period in weeks | Animals with skin tumours Obs. | Animals with skin tumours Exp. | Animals with infiltrating skin carcinomas Obs. | Animals with infiltrating skin carcinomas Exp. |
|---|---|---|---|---|
| 1–32 | 191 | 197·02 | 2 | 1·56 |
| 33–40 | 335 | 285·21 | 7 | 9·81 |
| 41–48 | 425 | 423·46 | 36 | 35·27 |
| 49–56 | 438 | 507·55 | 93 | 85·30 |
| 57–64 | 449 | 456·42 | 132 | 129·86 |
| 65–72 | 425 | 385·16 | 155 | 186·06 |
| 73–80 | 251 | 265·46 | 247 | 204·61 |
| 81–88 | 139 | 144·86 | 134 | 143·65 |
| 89–96 | 72 | 64·46 | 92 | 101·63 |
| 97–end | 26 | 21·40 | 51 | 51·25 |
| $\chi^2$ (7 d.f.) | 25·46 | | 17·20 | |
| $P$ | <0·001 | | <0·05 | |

statistically significant evidence that the overall shape of the time-to-tumour is not perfectly Weibull it is clearly a very good approximation. Despite a relative increase in tumour incidence rate between Weeks 32 and 96 by a factor of over 70 (TBA) and over 4000 (CBA) the observed numbers of tumours stay relatively close to those expected.

The goodness of fit to the Weibull distributions was investigated further by inspecting tabulations similar to Table IX for both individual treatment groups and whole experiments. The largest deviations were in Exp. 5, where 98 TBA were observed before Week 40, as against 50·2 expected, with a corresponding deficiency of observed TBA later on (Weeks 49–64 O = 74, E = 111·8), and in Exp. 7, where there was a marked deficiency in the first 32 weeks (O = 11, E = 35·5). In general, where these deviations occurred in an experiment, they were present for all treatment groups in that experiment, and there was therefore no suggestion that different $k$ and $w$ parameters should have been fitted for different types of treatment. Rather, the results suggest that something atypical had happened in these experiment at particular times, such as unusually poor visual inspection for tumours, or perhaps the presence of an infection. It was not possible to determine the actual identity of these occurrences but, as the principal conclusions are based on comparison within experiments rather than between experiments, the results obtained are unlikely to be severely biased by them.

*Definition and justification of the use of the tumorigenic ratio (TR)*

When assessing the relative activity of two different fractions (or of the same fractions from two different materials) it is useful to be able to do this on a weight-for-weight basis. However, for one to be able to say validly that a given weight of Fraction X produces an equivalent tumour response to T × that weight of Fraction Y (a TR of X/Y of T) for *any* given weight, it is necessary that the shape of the relationships between response and the log dose for the two fractions compared be parallel. Furthermore, it is convenient if the response variable can be chosen so that this relationship is linear. As there are theoretical reasons for expecting $b$ (and therefore WRP) to be proportional to a power of dose (Peto and Lee, 1973), choice of log $b$ as a response measurement was indicated, and Weibull mutiple regression analysis (*ibid.*) was therefore carried out to test the adequacy of the hypothesis that the relationship between log $b$ and log dose was linear and parallel for every treatment.

In this analysis, the parameters of the following models were estimated:

I $\log b_{ij} = \mu$
(No effect of treatment or dose)

II $\log b_{ij} = \mu + t_i$

(Effect of treatment only)

III $\log b_{ij} = \mu + t_i + q(\log \text{dose}_j)$

(Effect of treatment and linear effect of log dose)

IV $\log b_{ij} = \mu + t_i + r_j$
(Effect of treatment and dose but no interaction)

V $\log b_{ij} = \mu + s_{ij}$
(Effect of treatment, dose and interaction between treatment and dose)

where the $t_i$ represent differences from $\mu$ for the $i$th treatment and $r_j$ represent differences from $\mu$ for the $j$th dose level. The effects of treatment, dose and treatment + dose interaction were assessed by likelihood ratio tests, and the results of the analysis are summarized in Table X.

These results demonstrate that, though there are statistically significant departures from the hypothesis (Model III), as might be expected with such large amount of data carried out at different times, it explains a very great part of the observed variation between the groups. For TBA, 94% of the variation in response at different dose levels can be explained by the relationship $b \propto \text{dose}^{1\cdot37}$, and for CBA $b \propto \text{dose}^{1\cdot79}$ explains 98% of this variation.

Following further detailed inspection of the fit to the linear log $b$–log dose relationship (see next section) it was concluded that the values of TR presented in Tables II–VIII are a concise way of comparing different treatments which give, in virtually all cases, the very great part of the information relevant to the comparison.

The TR of two treatments $i_2$ and $i_1$ is formally calculated by:

$$\exp\,[(t_{i2} - t_{i1})/q]$$

using the results of Model III. The 95% confidence limits of the tumorigenic ratio T, were calculated by solving the quadratic equation (in U)

$$V^2 = (1\cdot96)^2 \text{ Var } V$$

where $V = qU - (t_2 - t_1)$, $U = \log T$ and the variances and covariances of $q$, $t_1$ and $t_2$ were obtained (asymptotically) from the multiple regression equations.

*Linearity of log dose-response relationship*

As noted in the discussion there are *a priori* reasons based on the findings of Davies *et al.* (1974) why the linear relationship assumed between log dose and log $b$ (or log WRP) might not hold at the highest dose levels tested. The detailed results of the multiple regression analysis were therefore examined to see where departures from the assumption occurred.

The main misfit occurred in SWS (C3) (see Table VIII) where the dose response was relatively steeper than that for other materials. The response at 65 mg was particularly low compared with that expected. The dose response for (R + P)G (T29) (see Table V) was also steeper that expected. For CBA, the main misfit was for C (T29) (see Table II) where the dose response was shallower than expected. However there was no

TABLE X.—*Results of Weibull Multiple Regression Analyses*

| Variables tested | Models compared | Degrees of freedom | Skin tumours $\chi^2$ | Skin tumours $P$ | Infiltrating skin carcinomas $\chi^2$ | Infiltrating skin carcinomas $P$ |
|---|---|---|---|---|---|---|
| Treatments | I v. II | 30 | 1579·28 | <0·001 | 805·18 | <0·001 |
| Linear effect of log dose (corrected for treatments) | II v. III | 1 | 488·47 | <0·001 | 234·23 | <0·001 |
| Non-linear effect of log dose (corrected for treatments and linear effect of log dose) | III v. IV | 6 | 32·58 | <0·001 | 5·15 | N.S. |
| Treatment × log dose interaction (corrected for treatments and log dose) | IV v. V | 17 | 49·55 | <0·001 | 38·32 | <0·01 |
| Total | I v. V | 54 | 2149·38 | — | 1082·88 | — |

marked evidence, as Davies' results would suggest, of a fall-off in response to SWS at 300 mg. The observed number of TBA on SWS at 300 mg (184) was somewhat below expectation (202·9), while those for fractions at 300 mg was somewhat above (O = 564, E = 534·8). While this is in the direction that Davies' results predicted, the magnitude of this difference is small and not statistically significant.